# Path planning algorithm for logistics autonomous vehicles at Cainiao stations based on multi-sensor data fusion

**DOI:** 10.1371/journal.pone.0321257

**Published:** 2025-05-20

**Authors:** Yan Chen

**Affiliations:** College of Transportation Management, Zhejiang Institute of Communications, Hangzhou, Zhejiang, China; Beijing Institute of Technology, CHINA

## Abstract

Efficient path planning and obstacle avoidance in a complex and dynamic environment is one of the key challenges of unmanned vehicle logistics distribution, especially in the logistics scene of Cainiao Station, which involves crowded communities and dynamic campus roads. In view of the shortcomings of existing methods in multi-sensor data fusion and path optimization, this paper proposes a path planning model based on multi-sensor image fusion, named DynaFusion-Plan. The model is able to provide an optimal path from the starting point to the target point in a complex environment, avoiding obstacles and realizing the smoothness and dynamic adjustment ability of the path. The model consists of three modules: the sensor data fusion module uses Convolutional Neural Networks (CNN) and Lidar-Inertial Odometry and Simultaneous Localization and Mapping (LIO-SAM) technology to build a high-precision dynamic environment map; the path planning module combines Artificial Potential Field (APF) and Deep Deterministic Policy Gradient (DDPG) algorithms to balance path length, smoothness, and obstacle avoidance capabilities; the decision and control module uses Model Predictive Control (MPC) and Long Short-Term Memory (LSTM) to achieve real-time path tracking and dynamic adjustment. Experimental results on TartanAir, NuScenes, and AirSim datasets show that DynaFusion-Plan significantly outperforms existing methods in key indicators such as path length (42.5 m vs. 48.7 m), path smoothness (κ=0.05 vs. κ=0.15), and obstacle avoidance success rate (98.7% vs. 85.4%), especially in complex dynamic environments. It shows strong adaptability and stability. This work provides an efficient and reliable solution for unmanned vehicle path planning in intelligent logistics scenarios, and lays the foundation for future optimization directions, such as lightweight model design and more real-world scenario verification.

## Introduction

With the rapid development of e-commerce and continuous advancements in intelligent logistics technologies, improving delivery efficiency has become a critical competitive indicator for logistics enterprises [1]. As an innovative terminal logistics model, Cainiao Stations provide efficient solutions for the transit and delivery of parcels by situating themselves in communities, schools, and similar environments [2–4]. However, in complex and dynamic scenarios—such as crowded residential areas and dynamic campus roads—how to efficiently plan the delivery paths for autonomous vehicles while ensuring safety and real-time performance remains a core challenge for Cainiao Station’s autonomous vehicle technology [5, 6].

Path planning for logistics autonomous vehicles is a key technology for achieving efficient deliveries. Its goal is to provide the optimal route from the starting point to the destination in complex environments, avoiding obstacles while ensuring smooth and dynamically adjustable paths [7–9]. The delivery scenarios at Cainiao Stations present unique challenges: (1) High path complexity, with dynamic obstacles such as pedestrians and vehicles, imposes stringent real-time requirements on path planning; (2) Diverse sources of environmental data, including multi-modal sensor inputs such as LiDAR, depth cameras, and IMUs, are essential for constructing high-precision environmental maps but simultaneously increase computational complexity [10, 11].

In recent years, the development of multi-sensor fusion and deep learning technologies has offered new solutions to path planning challenges. Multi-sensor fusion significantly enhances environmental modeling accuracy and dynamic environment perception by integrating data from LiDAR, depth cameras, and IMUs [12]. Simultaneously, reinforcement learning methods based on deep learning, such as Deep Q-Networks (DQN) and Deep Deterministic Policy Gradient (DDPG), enable real-time optimization and adjustment of paths in dynamic environments [13, 14]. However, effectively integrating multi-sensor fusion and deep learning technologies to meet the high-precision, real-time, and robustness requirements of path planning in Cainiao Station logistics scenarios remains a pressing technical challenge [15–17].

To solve the above problems, this paper proposes DynaFusion-Plan, a path planning algorithm framework based on multi-sensor data fusion. The framework consists of three modules: sensor data fusion module, using Convolutional Neural Networks (CNN) and Lidar Inertial Odometry and Simultaneous Localization and Mapping (LIO-SAM) technology to build high-precision environment map to perceive dynamic changes in distribution scenario; path planning module, combining artificial potential field method (APF) and deep deterministic strategy gradient algorithm (DDPG) to realize path optimization and obstacle avoidance in dynamic environment; decision and control module to complete path tracking and dynamic adjustment of unmanned vehicles based on model prediction and control (MPC) and long-and short-term memory network (LSTM).

The main contributions of this paper are as follows:

- A dynamic path planning algorithm framework that integrates multi-sensor fusion and deep learning, specifically tailored to the requirements of Cainiao Station logistics autonomous vehicles.
- Significant improvements in environmental modeling accuracy and path planning robustness through the deep integration of multi-sensor data fusion and reinforcement learning technologies.
- Validation of the proposed framework’s effectiveness and adaptability in complex delivery scenarios using both real-world and simulated datasets.

## Related works

### Multi-sensor fusion for complex environments

Multi-sensor fusion is a core technology for achieving high-precision environmental perception, particularly in complex and dynamic environments where it significantly enhances system robustness and accuracy. In practical applications, autonomous vehicles rely on various sensors, such as LiDAR, depth cameras, and Inertial Measurement Units (IMU), to obtain environmental information [18, 19]. LiDAR provides high-precision 3D point cloud data, making it ideal for obstacle detection and environmental modeling [20, 21]. Depth cameras capture visual information, aiding in the identification of dynamic objects and providing rich environmental textures. IMUs supply motion state and attitude data for the vehicle [22, 23]. However, individual sensors have inherent limitations. For instance, LiDAR performs poorly in low-light conditions, depth cameras are prone to failure under strong lighting or reflective surfaces (glass or water), and IMUs accumulate errors over time [24]. By fusing data from multiple sensors, these shortcomings can be mitigated, resulting in more stable and accurate environmental perception.

Currently, data fusion methods based on Kalman Filter (KF) and Extended Kalman Filter (EKF) are widely used in sensor information integration for autonomous vehicles, particularly excelling in Simultaneous Localization and Mapping (SLAM). EKF effectively fuses data from LiDAR and IMUs, providing precise estimates of robot posture and position tracking [25]. Additionally, graph optimization methods, such as Graph-SLAM, transform sensor data into graph structures and utilize optimization algorithms to further improve map accuracy and stability [26]. These fusion techniques have been successfully applied in various domains, including autonomous driving, robotic navigation, and augmented reality [27]. Despite their effectiveness in static environments, existing fusion methods face significant challenges in complex dynamic scenarios. Handling dynamic obstacles, rapidly changing environments, and the effective integration of multi-modal data remain pressing issues [28]. To address these challenges, this study integrates Convolutional Neural Networks (CNNs) with LIO-SAM technology within the proposed multi-sensor image fusion path planning framework. CNNs are used to extract image features, complementing LIO-SAM for efficient modeling of complex environments. This integration enables precise path planning and robust obstacle avoidance capabilities.

### Deep learning in path planning and environmental perception

Deep learning has achieved remarkable breakthroughs in path planning, particularly in the field of Reinforcement Learning (RL). Traditional path planning algorithms, such as A* and Dijkstra, typically rely on predefined environmental models and static obstacle information, making them inadequate for addressing real-time changes and unpredictability in complex dynamic environments [29]. Reinforcement Learning, especially Deep Q-Networks (DQN) and Deep Deterministic Policy Gradient (DDPG), learns strategies through interaction with the environment, enabling real-time optimization of paths, collision avoidance, and effective handling of dynamic obstacles [30, 31]. However, these methods face challenges such as lengthy training times and slow model convergence. In complex environments, balancing training efficiency with the real-time requirements of path planning remains a significant research challenge [32]. Deep reinforcement learning (DRL) models have shown significant promise in path planning, particularly in dynamic and unpredictable environments [33]. However, these models are often susceptible to sudden changes in the environment, such as unexpected obstacles or rapid environmental shifts, which can cause fluctuations in their stability and reliability. One primary reason for this instability is the slow response to new, unseen situations during training, especially when the model encounters states outside the training distribution. Additionally, the convergence rate of DRL models can be hindered by complex, high-dimensional state spaces and the challenges associated with exploring large action spaces effectively. These issues can lead to erratic behavior and suboptimal performance, particularly in real-time applications where fast and stable decisions are critical.

In the realm of environmental perception, deep learning has also demonstrated immense potential, particularly in object detection and 3D environment modeling. CNN-based object detection algorithms, such as YOLO and Faster R-CNN, are widely applied for detecting dynamic obstacles. These algorithms can identify and locate dynamic objects like pedestrians and vehicles in real time within complex environments [34]. However, existing deep learning methods still face challenges in efficiently fusing data from multiple sensors, such as LiDAR, cameras, and IMUs, to improve perception accuracy. Deep learning methods for processing point cloud data, such as PointNet and PointRCNN, achieve high accuracy in 3D environment reconstruction and obstacle detection but have substantial computational overhead, limiting their applicability in real-time scenarios [35]. Furthermore, in highly dynamic and complex logistics environments, such as those at Cainiao Stations, managing rapidly changing surroundings and numerous non-static obstacles remains a significant challenge [36].

Recently, decision path planning methods based on distributed reinforcement learning have been developed significantly. By sensing the uncertainty of the environment, these methods are able to optimize the decision process and improve the accuracy and stability of path planning in a dynamically changing environment [37]. In addition, the proposal of the space-time constrained path planning algorithm makes the path planning more efficient and real-time in complex traffic flow and intersection road conditions. Such algorithms can adapt to the mixed traffic flow and optimize the planning results, providing new ideas to solve the problems of real-time and feasibility [38]. With the gradual deepening of the application of unmanned vehicle technology in the transportation system, the relevant research also promotes the innovation of path planning algorithm, which provides an important reference for the practical application and technical development of unmanned vehicle [39].

To address these challenges, this study integrates CNNs with LIO-SAM, enhancing accuracy in static environment modeling. Additionally, DDPG is utilized to optimize path planning and real-time adjustments in dynamic environments. This approach effectively addresses path planning challenges in complex dynamic scenarios, such as those encountered in Cainiao Stations, demonstrating improved accuracy and robustness, particularly in handling dynamic obstacles and real-time feedback adjustments.

### Development and application of intelligent logistics technology

With continuous advancements in technology, intelligent logistics has undergone rapid development in recent years and has been widely applied globally. Path optimization is one of the core technologies in intelligent logistics systems, aiming to optimize the travel paths of logistics vehicles while ensuring safety, thereby improving delivery efficiency [40, 41]. Traditional path planning methods, such as A* and Dijkstra algorithms, have been extensively used in relatively simple logistics scenarios. However, as logistics environments become increasingly complex—characterized by dynamic obstacles, variable traffic conditions, and real-time delivery demands—the limitations of traditional algorithms have become apparent [42]. In recent years, intelligent algorithms, such as Genetic Algorithms (GA), Ant Colony Optimization (ACO), and Reinforcement Learning (RL), have emerged as promising solutions for path optimization. These methods can handle more complex constraints and enhance the real-time performance and robustness of path planning. Beyond path optimization, intelligent scheduling and delivery are also critical components of intelligent logistics systems [43, 44]. Data-driven scheduling models leverage big data, machine learning, and optimization algorithms to automate logistics task scheduling, thereby improving the overall efficiency and responsiveness of transport networks [45].

Despite significant progress in intelligent logistics technologies, numerous challenges persist in practical applications. First, the issues of real-time performance and adaptability in path planning under dynamic environments remain unresolved. Logistics fleets operating in complex urban settings often face unpredictable traffic congestion, sudden obstacles, and weather changes, posing stringent requirements on the real-time and adaptive capabilities of path planning algorithms [46]. Second, the integration of path planning and control technologies for autonomous vehicles within intelligent logistics systems remains a critical challenge [47]. While multi-sensor fusion and deep learning methods have been widely applied in the perception and decision-making processes of autonomous vehicles, achieving precise and efficient path planning and control in large-scale, dynamically changing environments remains a bottleneck in technological development [42]. To address these challenges, this study combines the strengths of deep learning and reinforcement learning to enable efficient path planning and real-time obstacle avoidance in dynamic environments. By incorporating multi-sensor fusion, the proposed approach enhances perception accuracy and system robustness, effectively tackling the complex challenges faced in intelligent logistics applications.

## Methods

### Overview of the model architecture

**Fig 1:**
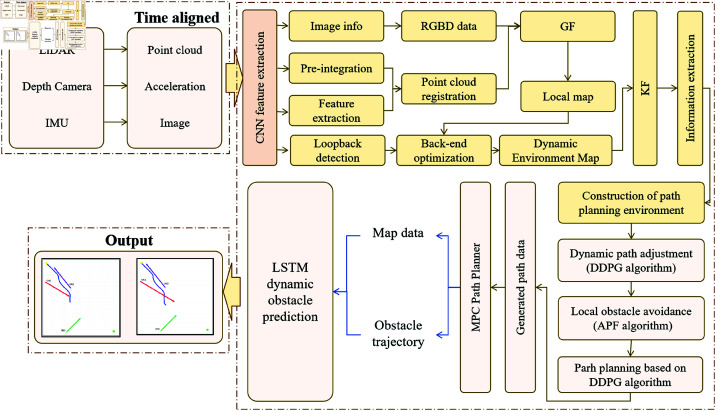
DynaFusion-Plan Model Overall Architecture Flowchart: Illustrates the collaborative workflow among the multi-sensor data fusion, path planning, and control decision-making modules, as well as their functional framework for achieving path planning and obstacle avoidance in complex dynamic environments.

The proposed DynaFusion-Plan model is designed to address the path planning challenges of logistics autonomous vehicles at Cainiao Stations in complex dynamic environments. The framework comprises three primary modules: the Sensor Data Fusion Module, the Path Planning Module, and the Decision and Control Module. Fig 1 illustrates how these modules collaboratively operate to achieve efficient and precise path planning with dynamic obstacle avoidance.

As shown in Fig 1, the Sensor Data Fusion Module serves as the foundation of the system, responsible for building a high-precision environmental model and providing real-time perception by fusing data from various sensors such as LiDAR, depth cameras, and IMUs. By combining CNNs with LIO-SAM (LiDAR-Inertial Odometry and Simultaneous Localization and Mapping), this module extracts features from image data using deep learning techniques and integrates them with LiDAR point cloud data. LIO-SAM enables real-time modeling of dynamic environments. The sensor data fusion not only enhances the accuracy of the environmental model but also ensures a rapid response to dynamic environmental changes, providing reliable input data to the Path Planning Module, which is crucial for subsequent accuracy and real-time performance.

Building on this foundation, the Path Planning Module generates the optimal path for the autonomous vehicle based on environmental perception. Traditional path planning algorithms, such as A* and Dijkstra, typically assume a static environment, making them unsuitable for handling dynamic obstacle changes. This study combines the APF method with DDPG to effectively address path planning in dynamic environments. APF efficiently handles local obstacle avoidance, while DDPG optimizes the global path to ensure smoothness and safety. The combination of these methods enables the model to flexibly adjust and optimize paths even in scenarios with frequently changing dynamic obstacles.

Next, the Decision and Control Module is responsible for executing path tracking and making dynamic adjustments based on the results of the Path Planning Module, ensuring that the autonomous vehicle travels stably and accurately. This module employs MPC to perform path tracking and decision-making, complemented by LSTM networks to predict the future trajectories of obstacles and adjust the path accordingly. MPC forecasts the path over a future time horizon and optimizes control inputs to achieve precise path following. Meanwhile, LSTM enables the model to anticipate the movement of dynamic obstacles, proactively identifying potential collision points to ensure safe navigation in complex environments.

These three modules work together through a tightly integrated mechanism. The Sensor Data Fusion Module provides high-precision environmental information to the Path Planning Module, which generates real-time optimized paths and forwards the results to the Decision and Control Module for execution. The flow of information and feedback among the modules ensures continuity and stability in path planning and control. This highly collaborative design allows the DynaFusion-Plan model to tackle path planning challenges in complex dynamic environments, ensuring efficient and precise obstacle avoidance and tracking in Cainiao Station logistics scenarios.

Fig 1 clearly illustrates the overall architecture of the model and the relationships between the modules. The workflow depicted in the figure highlights the process from sensor data acquisition to path planning and control execution, emphasizing the collaborative roles and information flow among the modules. This modular design not only enhances system flexibility and scalability but also enables the solution to adapt to diverse application scenarios, facilitating efficient deployment and optimization in complex logistics environments such as Cainiao Stations.

### Sensor data fusion module

The Sensor Data Fusion Module is a core component of the DynaFusion-Plan model, responsible for constructing a high-precision environmental map by fusing data from various sensors, and for real-time perception and updating of the dynamic environment. Through the operation of this module, the system achieves accurate environmental awareness, providing reliable input data for subsequent path planning and control. In complex dynamic environments, the effective fusion of data from LiDAR, depth cameras, and IMUs to rapidly and precisely construct an environmental model is the prerequisite for ensuring the accuracy and safety of path planning [48, 49].

**Fig 2:**
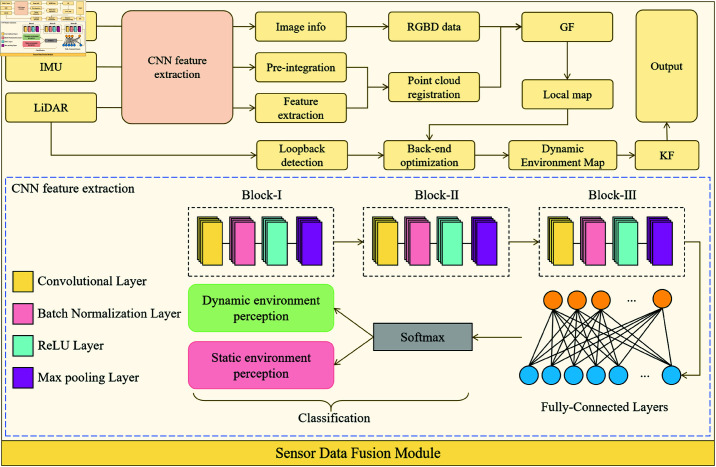
Sensor Data Fusion Module: Includes the overall workflow of multi-sensor data acquisition (LiDAR, depth camera, IMU), preprocessing, CNN feature extraction, LIO-SAM data fusion, and real-time dynamic environment updates.

As shown in Fig 2, the design architecture of the Sensor Data Fusion Module includes several submodules, each responsible for the acquisition, preprocessing, feature extraction, and fusion of different sensor data. The entire workflow of the module consists of three main steps: the acquisition and preprocessing of sensor data, the extraction of image features using CNN and fusion with LIO-SAM data, and the real-time update and noise elimination of the dynamic environment. This process is realized through the coordination of several interconnected submodules, ensuring the complete data processing mechanism from acquisition to fusion, processing, and updating.

In the first stage, the system collects environmental data through integrated sensors, such as LiDAR, depth cameras, and IMUs. LiDAR provides high-precision 3D point cloud data, the depth camera captures visual features of the environment, and the IMU provides information on the vehicle’s acceleration and angular velocity. Due to the large differences in data formats and characteristics among these sensors, preprocessing is required. The main tasks in preprocessing include noise removal of the point cloud data, cropping and normalization of depth images, and drift correction of IMU data. Noise removal of point cloud data can be achieved using Gaussian filtering [50], as shown below:

$$P^{\prime }=\frac{1}{N}\sum_{i=1}^{N}P_{i}\cdot w_{i}$$ (1)

where $P^{\prime }$ represents the denoised point cloud data, *P*_*i*_ is each LiDAR point, *w*_*i*_ is the weight coefficient, and *N* is the number of sampling points.

After preprocessing, the second stage involves extracting image features using CNNs and fusing these features with LiDAR point cloud data to generate a high-precision environmental model. The image data captured by the depth camera is processed by the CNN, which extracts semantic information about the environment, such as static obstacles and dynamic objects. This process can be represented by the following convolution operation formula:

$$y_{i}=\sum_{j}W_{ij}x_{j}+b_{i}$$ (2)

where *y*_*i*_ is the feature output after the convolution operation, *x*_*j*_ represents the pixel values of the input image, *W*_*ij*_ is the convolution kernel, and *b*_*i*_ is the bias.

The features extracted by the CNN are then fused with LiDAR point cloud data through LIO-SAM. LIO-SAM utilizes IMU data for attitude estimation and combines it with LiDAR point cloud data for environmental modeling. The optimization process of LIO-SAM is as follows:

$${\hat{x}}_{k}=\underset{x}{\arg\min}\sum_{i=1}^{N}\left\|z_{i}-\right.{\left.h(x,u_{i})\right\|}_{{\text{Σ}}_{i}}^{2}$$ (3)

Where ${\hat{x}}_{k}$ is the optimized state estimate, *z*_*i*_ is the measurement from the *i*-th sensor, *h*(*x*,*u*_*i*_) is the state prediction function, *u*_*i*_ is the control input or input parameter for the *i*-th sensor, $\Sigma_{i}$ is the measurement error covariance matrix for the *i*-th sensor, and *N* is the total number of data points.

The LIO-SAM algorithm, through joint optimization of LiDAR point cloud data and IMU data, provides accurate state estimation and map building capabilities, ensuring that the fused environmental model effectively reflects changes in the dynamic environment.

In the real-time updating process, the Sensor Data Fusion Module dynamically updates the environmental model and eliminates noise in real-time. In this study, Kalman Filtering (KF) is used for noise elimination in sensor data:

$${\hat{x}}_{k}=A{\hat{x}}_{k-1}+Bu_{k}+K(z_{k}-H{\hat{x}}_{k-1})$$ (4)

where ${\hat{x}}_{k}$ is the state estimate at the current moment, *A* and *B* are the state transition matrix and control matrix of the system model, *K* is the Kalman gain, *z*_*k*_ is the actual observation value, and *H* is the observation matrix. Through Kalman filtering, the system can continuously correct the environmental model in a dynamic environment, eliminating the impact of noise and ensuring efficient fusion and accuracy of sensor data.

The Sensor Data Fusion Module successfully realizes high-precision environmental perception and real-time updates through the collaborative work of multiple processing steps and algorithms. This module provides reliable environmental input to the Path Planning Module, effectively supporting the DynaFusion-Plan model’s path planning and obstacle avoidance capabilities in dynamic, complex environments.

### Path planning module

The Path Planning Module is one of the core components of the DynaFusion-Plan model, with the primary task of planning the optimal path for the unmanned vehicle in dynamic environments. This module combines the advantages of two techniques: the APF method and the DDPG algorithm. APF is used for local obstacle avoidance and path optimization [51], while DDPG optimizes the global path through reinforcement learning, ensuring that the unmanned vehicle can navigate safely and efficiently in complex and dynamic environments, thereby improving the path planning efficiency, smoothness, and obstacle avoidance capabilities [52, 53].

**Fig 3:**
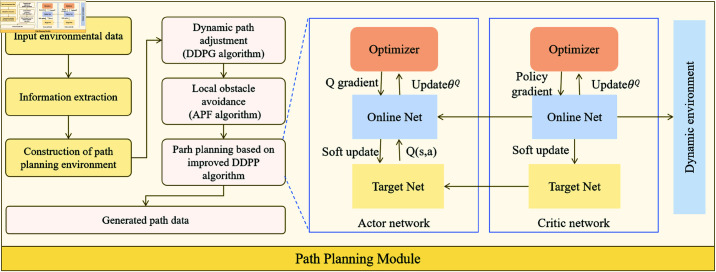
Path Planning Module: Includes the full workflow of global path generation, local obstacle avoidance (APF), dynamic path adjustment (DDPG), and path smoothing optimization, as well as the collaborative mechanisms between modules.

As shown in Fig 3, the overall architecture of the Path Planning Module includes several key steps: global path generation, local obstacle avoidance and path optimization, and dynamic path adjustment based on deep reinforcement learning. Global path generation uses the classical algorithm, which generates an initial path based on the environmental map and the positions of the starting point and the target point. The global path typically considers major static obstacles in the environment, but it does not account for real-time dynamic obstacles.

f(n)=g(n)+h(n) (5)

where f(n) is the total cost of node *n*, g(n) is the cost from the start to node *n*, and h(n) is the estimated cost from node *n* to the target point. This way, A* can quickly find the shortest path from the start point to the target point over a large area.

After the global path is generated, the Artificial Potential Field (APF) method is employed for local obstacle avoidance. APF generates attractive and repulsive forces for the target point and obstacles, respectively, guiding the unmanned vehicle to avoid obstacles and move towards the target point. Let the position of the target point be $P_{\text{target}}$ and the position of the obstacle be $P_{\text{obs}}$, then the attractive force of the target point and the repulsive force of the obstacle are as follows:

$$F_{\text{target}}=-k_{\text{attraction}}\cdot (P-P_{\text{target}})$$ (6)

$$F_{\text{obs}}=k_{\text{repulsion}}\cdot \frac{1}{\|P-P_{\text{obs}}{\|}^{2}}$$ (7)

where $k_{\text{attraction}}$ and $k_{\text{repulsion}}$ are the proportional coefficients for the attractive and repulsive forces, *P* is the current position of the unmanned vehicle, and $\left\|P\;-\;P_{\text{obs}}\right\|$ is the distance from the vehicle to the obstacle. Through the superposition of these forces, APF can guide the unmanned vehicle to avoid obstacles and move towards the target point.

However, in complex environments, the path generated by APF may not always be optimal or smooth. Therefore, we introduce the DDPG algorithm to optimize the global path. DDPG uses deep reinforcement learning with continuous action spaces, learning the optimal path planning strategy through interaction with the environment, enabling the unmanned vehicle to learn how to adjust the path in real-time in a changing environment. Its update formula is as follows:

$$Q^{\prime }=r+\gamma Q(s^{\prime },a^{\prime })$$ (8)

where $Q^{\prime }$ is the new state value, *r* is the reward, γ is the discount factor, and $Q(s^{\prime },a^{\prime })$ is the Q-value of the next state-action pair. During training, DDPG optimizes the path selection strategy by maximizing cumulative rewards and improves path planning efficiency and accuracy through exploration-exploitation mechanisms.

Whenever the unmanned vehicle receives new sensor data, the path planning module recalculates and adjusts the path based on environmental changes, ensuring that the vehicle can avoid obstacles and reach the target point in real-time. The path adjustment process is as follows:

$$\Delta \theta_{t}=\alpha \cdot \frac{\partial Q}{\partial \theta_{t}}$$ (9)

where $\Delta \theta_{t}$ is the path adjustment, α is the learning rate, and $\frac{\partial Q}{\partial \theta_{t}}$ is the gradient of the Q-value with respect to the path parameter $\theta_{t}$.

However, the path may become unsmooth due to real-time adjustments, which increases the control difficulty of the unmanned vehicle. Therefore, a Quadratic Programming (QP) algorithm is used to balance the smoothness of the path and obstacle avoidance capability. By minimizing the curvature of the path, smoother paths can be generated while maintaining obstacle avoidance.

$$\min_{𝐏}\int_{0}^{T}\left(\lambda_{1}\|P^{\prime \prime }(t){\|}^{2}+\lambda_{2}\|P^{\prime }(t)-𝐯(t){\|}^{2}\right)dt$$ (10)

where $P^{\prime \prime }(t)$ is the second derivative of the path (i.e., curvature), $P^{\prime }(t)$ is the velocity of the path, 𝐯(t) is the target velocity, and $\lambda_{1}$ and $\lambda_{2}$ are the weight coefficients for smoothness and tracking error.

Through the above processes, the path planning module can generate efficient and smooth paths in complex dynamic environments, with real-time dynamic adjustments and optimizations. This effectively solves the path planning problem in complex environments, allowing the system to handle dynamic obstacle changes and improve the real-time performance and robustness of path planning while ensuring smooth paths.

### Decision and control module

The Decision-Making and Control Module is a crucial component of the DynaFusion-Plan model, ensuring that the unmanned vehicle can accurately follow its path and adjust in real-time within dynamic environments. This module combines MPC and LSTM networks to achieve both path tracking control and dynamic obstacle prediction and avoidance. MPC is used for real-time path tracking optimization [54], while LSTM learns the motion trajectories of obstacles and predicts their future positions, providing decision support for path adjustments [15, 55].

**Fig 4:**
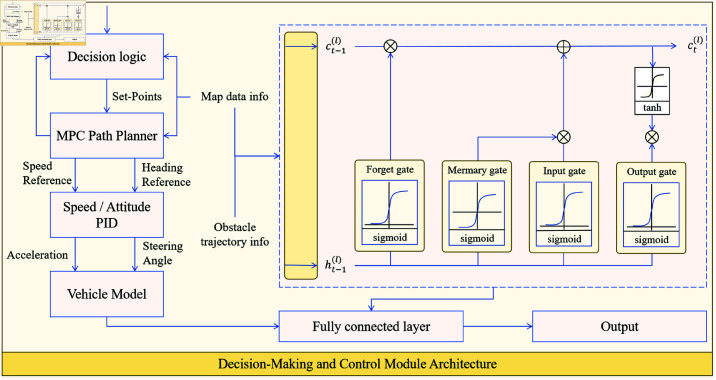
Decision-Making and Control Module Architecture: This includes the collaborative workflow of path tracking and real-time optimization based on MPC, along with dynamic obstacle trajectory prediction and path adjustment based on LSTM.

As shown in Fig 4, the design architecture of the Decision-Making and Control Module consists of four main sub-modules: path tracking control, dynamic obstacle prediction, path adjustment, and optimization. Path tracking control is implemented through MPC for precise control; obstacle trajectory prediction is done using LSTM to forecast the future positions of obstacles; and path adjustment and optimization are based on the aforementioned predictions, making real-time control adjustments to ensure that the unmanned vehicle can move smoothly while avoiding potential collisions. The sub-modules collaborate through tightly integrated data flows and feedback mechanisms, providing a highly integrated solution for path tracking and dynamic environment adaptation.

In this section, path tracking control is key to ensuring the unmanned vehicle follows the planned path with high accuracy. MPC optimizes the control inputs to minimize path tracking errors, achieving precise motion control for the vehicle.

$$\min_{𝐮}\sum_{t=0}^{N}\left(\|{𝐱}_{t}-{𝐱}_{\text{ref}}(t){\|}_{Q}^{2}+\|{𝐮}_{t}{\|}_{R}^{2}\right)$$ (11)

where ${𝐱}_{t}$ is the vehicle’s state at time *t*, ${𝐱}_{\text{ref}}(t)$ is the desired state on the reference path, ${𝐮}_{t}$ is the control input (such as acceleration or steering angle), *Q* and *R* are weight matrices, and *N* is the prediction time step. By optimizing this objective function, MPC generates the optimal control input, making the vehicle’s state as close as possible to the desired path.

In MPC, it is usually necessary to predict future states to optimize control inputs. This process recursively calculates the optimal control inputs to ensure smooth tracking of the vehicle in dynamic environments.

$${𝐱}_{t+1}=f({𝐱}_{t},{𝐮}_{t})$$ (12)

where ${𝐱}_{t+1}$ represents the vehicle’s state at time t+1, and $f({𝐱}_{t},{𝐮}_{t})$ is the state transition function of the system, representing the vehicle’s dynamic model.

Path tracking control relies not only on the current environmental state but also on predicting the future positions of obstacles. In this module, LSTM is used to predict the positions of dynamic obstacles in future time steps, providing decision support for path adjustments. The core of the LSTM model is its gating structure, which effectively retains long-term dependency information.

$$i_{t}=\sigma (W_{i}[h_{t-1},x_{t}]+b_{i})\;$$ (13)

$$f_{t}=\sigma (W_{f}[h_{t-1},x_{t}]+b_{f})\;$$ (14)

$$h_{t}=o_{t}\cdot \tanh(c_{t})\;$$ (15)

where *i*_*t*_, *f*_*t*_, and *o*_*t*_ are the activation functions for the input gate, forget gate, and output gate, respectively, *c*_*t*_ is the cell state, *h*_*t*_ is the output of the LSTM, *x*_*t*_ is the input data (current position of the obstacle), and *W*_*i*_, *W*_*f*_, and *b*_*i*_, *b*_*f*_ are the parameters of the LSTM.

Based on the obstacle prediction results from LSTM, the path adjustment module can perform real-time optimization of the original path, ensuring that the unmanned vehicle avoids regions where collisions might occur in the future. The path adjustment goal, which combines obstacle prediction information, MPC control results, and the current state, generates a smooth and safe path.

$${𝐏}_{\text{adjusted}}={𝐏}_{\text{original}}+\Delta 𝐏$$ (16)

where ${𝐏}_{\text{adjusted}}$ is the adjusted path, ${𝐏}_{\text{original}}$ is the original path, and Δ𝐏 is the path adjustment. The path adjustment Δ𝐏 is determined by both the obstacle prediction information and the current control input.

To make the adjusted control inputs smoother and feasible, the following objective function is used for modeling:

$$\min_{𝐮}\sum_{t=0}^{N}\left(\|{𝐮}_{t}-{𝐮}_{\text{ref}}(t){\|}_{R}^{2}+\lambda \|{𝐱}_{t}-{𝐱}_{\text{ref}}(t){\|}_{Q}^{2}\right)$$ (17)

where ${𝐮}_{\text{ref}}(t)$ is the reference control input, λ is the smoothing weight, and other symbols are as defined previously. This optimization objective aims to balance the smoothness of the control input with the path tracking accuracy, ensuring that the unmanned vehicle remains stable and safe during adjustments.

In path tracking control, the goal of Model Predictive Control (MPC) is to minimize path tracking error while optimizing control inputs to ensure the autonomous vehicle follows the reference path precisely. However, optimizing only for path tracking accuracy may lead to sharp changes in the path, which increases the complexity and instability of the vehicle’s control. To balance path accuracy and stability, we introduce a smoothness weight term aimed at reducing the magnitude of path changes, making the path smoother, thus improving the feasibility and safety of control. The objective function in (Eq 17) adds a smoothness term to (Eq 11), specifically by introducing weights for the smoothness of control inputs and path tracking accuracy, λ, to achieve smooth path adjustment and optimization. This improvement is based on quadratic programming theory, aiming to optimize the system’s trajectory, reduce unnecessary path twists and fluctuations in control inputs, thereby ensuring efficient operation and stability of the autonomous vehicle in dynamic environments. In the MPC optimization process, the balance between smoothness and path tracking accuracy is achieved by adjusting the weight λ, enabling the path to avoid collisions while maintaining reasonable smoothness.

Through the collaboration of MPC and LSTM, the decision and control module enables real-time path tracking, dynamic obstacle prediction, and dynamic path adjustment in dynamic environments, ensuring that the unmanned vehicle can follow paths and make real-time adjustments in complex, dynamic environments. This provides strong support for the application of the DynaFusion-Plan model in complex logistics scenarios, such as in Cainiao stations.

## Experiment

### Datasets

In this study, three publicly available datasets—TartanAir Dataset, NuScenes Dataset, and AirSim Dataset—are selected to validate the path planning and obstacle avoidance capabilities of the DynaFusion-Plan model in dynamic environments. These datasets cover different sensor configurations, environmental scenarios, and complex dynamic obstacles, providing comprehensive testing grounds for the model.

The TartanAir Dataset is a synthetic dataset designed for research on visual and LiDAR data fusion. It includes a variety of complex dynamic scenarios, making it ideal for evaluating SLAM and path planning algorithms. The dataset provides RGB images, depth images, LiDAR point clouds, and IMU data, allowing for comprehensive testing of multi-sensor fusion accuracy and path planning performance [56]. The choice of TartanAir is based on its rich set of dynamic obstacles and diverse terrain scenarios, which are crucial for testing the model’s robustness in dynamic environments. Additionally, the multimodal data provided by the dataset enables validation of the DynaFusion-Plan model’s effectiveness in multi-sensor fusion.

The NuScenes Dataset is a large-scale autonomous driving dataset that contains real-time data from multiple sensors, including LiDAR, radar, cameras, and IMU. The scenes in the dataset include urban roads, highways, and parking lots, with detailed annotations of dynamic obstacles, making it particularly suitable for evaluating path planning and dynamic obstacle avoidance algorithms [57]. The advantage of the NuScenes dataset lies in its extensive urban road scenarios and dynamic obstacles, making it ideal for validating the DynaFusion-Plan model’s application in complex urban traffic environments. Its diverse sensor configurations also support multimodal data fusion and dynamic environment perception validation.

The AirSim Dataset, developed by Microsoft as an open-source simulator, is specifically designed for autonomous driving and drone research. This dataset supports various sensor types, such as RGB cameras, depth cameras, LiDAR, and IMU, and allows for the generation of different obstacles and environmental changes within the simulation. It is suitable for testing reinforcement learning, path planning, and control algorithms [58]. The advantage of the AirSim dataset lies in its highly controllable simulation environment, which can be used to design a wide variety of test scenarios and simulate complex dynamic environments. This makes it especially well-suited for validating the DynaFusion-Plan model’s capabilities in reinforcement learning and dynamic path adjustment.

Table 1 summarizes the key information of these three datasets, including sensor configurations, environmental scenes, and data types. The characteristics of each dataset provide distinct dimensions of support for validating the model in this paper.

**Table 1:** Key Information of TartanAir, NuScenes, and AirSim Datasets.

Dataset	Sensor Configuration	Scene Types	Main Data Types	Applicability
TartanAir	LiDAR, Depth Camera, IMU	Synthetic data, dynamic obstacles, mountainous, urban, etc.	RGB images, depth images, LiDAR point clouds, IMU	Testing multi-sensor fusion, SLAM, dynamic scene evaluation
NuScenes	LiDAR, Radar, Camera, IMU	Urban roads, highways, parking lots, etc	LiDAR point clouds, radar data, video streams, IMU	Path planning and obstacle avoidance in complex urban environments
AirSim	LiDAR, RGB Camera, Depth Camera, IMU	Adjustable simulation scenes	RGB images, depth images, LiDAR point clouds, IMU	Reinforcement learning and path planning in simulation environments

By integrating these three datasets, this paper is able to comprehensively validate the path planning and obstacle avoidance capabilities of the DynaFusion-Plan model across different environments, ensuring the model’s robustness and real-time responsiveness in dynamic settings. The diversity and complexity of these datasets provide ample support for our experiments, making the results more representative and broadly applicable.

### Experimental setup and metrics

This experiment combines both simulation and real-world data for evaluation. The experimental environment is built on a standard computing platform, and the model is trained and evaluated using the corresponding training configurations. Table 2 summarizes the environmental setup and key parameters for model training, including hardware configurations, hyperparameters for optimization algorithms, network architecture, and other critical settings.

**Table 2:** Summary of Experimental Setup, which presents the hardware configuration, model training parameters, and key settings for the path planning algorithm.

Parameter Category	Setting	Description
Hardware Environment	Intel i7-10700K CPU	Hardware configuration used for accelerating the training process
Operating System	Ubuntu 20.04	Batch Size:32; Training Epochs:50; Network Architecture: CNN (ResNet18) + LSTM (2 layers, 128 units per layer)
Deep Learning Framework	Reinforcement Learning Parameters (DDPG)	Target network update frequency: 1000 steps; Discount factor: 0.99; Experience replay pool size: 1,000,000
	Path Smoothing Optimization (MPC)	Time step: 0.1 seconds; Prediction horizon: 10 seconds; Weight coefficients (Q, R): 5, 1
	Environment Perception Fusion (LIO-SAM)	LiDAR resolution: 0.5°; IMU frequency: 200Hz
	Path Planning Algorithm (APF)	Attraction coefficient: 1.0; Repulsion coefficient: 2

These settings ensure that the experiment thoroughly tests the performance of the DynaFusion-Plan model across various aspects.

### Evaluation metrics

This paper designs several evaluation metrics that cover aspects such as path planning quality, control performance, and the real-time responsiveness of the model, thus providing a comprehensive assessment of the performance of the DynaFusion-Plan model.

The path planning metrics mainly include path length (*L*), smoothness (κ), and obstacle avoidance success rate ($R_{\text{OAS}}$). A shorter path length indicates higher efficiency in path planning. Smoothness is typically evaluated by the curvature of the path, where smaller curvature values correspond to smoother paths. The obstacle avoidance success rate is defined as the ratio of successfully avoided path points to the total number of path points, reflecting the model’s ability to avoid obstacles in a dynamic environment.

$$L=\sum_{i=1}^{N}\|{𝐩}_{i}-{𝐩}_{i-1}\|$$ (18)

Where ${𝐩}_{i}$ and ${𝐩}_{i-1}$ represent two adjacent points on the path, and *N* is the total number of points on the path.

$$\kappa =\frac{\left\|{𝐩}^{\prime }(t)\times {𝐩}^{\prime \prime }(t)\right\|}{\|{𝐩}^{\prime }(t){\|}^{3}}$$ (19)

Where ${𝐩}^{\prime }(t)$ and ${𝐩}^{\prime \prime }(t)$ represent the velocity and acceleration of the path at time *t*, respectively.

$$R_{\text{OAS}}=\frac{\text{Number of successfully avoided path points}}{\text{Total number of path points}}$$ (20)

The control performance metrics mainly include path tracking error ($e_{\text{track}}$) and decision delay (D). Path tracking error is measured by calculating the distance from the vehicle’s current position to the nearest point on the path. Smaller errors mean the model can more accurately follow the planned path. Decision delay is the time taken from sensor data acquisition to path adjustment instruction generation, measuring the system’s responsiveness to changes in the dynamic environment. A lower decision delay means the system can adapt more quickly to environmental changes.

$$e_{\text{track}}=\min_{i}\|{𝐩}_{i}-{𝐩}_{\text{current}}\|$$ (21)

Where ${𝐩}_{i}$ is a point on the path, and ${𝐩}_{\text{current}}$ is the current position of the unmanned vehicle.

$$\text{D}=t_{\text{decision}}-t_{\text{sensor}}$$ (22)

Where $t_{\text{decision}}$ is the time of the path planning decision, and $t_{\text{sensor}}$ is the time of sensor data acquisition.

### Comparative experiment

In the comparative experiments, the DynaFusion-Plan model is evaluated against several state-of-the-art path planning algorithms, including A*, Dijkstra, RRT, Bi-RRT, PRM, and Hybrid-SLAM. The comparison focuses on five key metrics: path length, smoothness, obstacle avoidance success rate, path tracking error, and decision delay. The experimental results are summarized in Table 3. The results demonstrate that the DynaFusion-Plan model achieves superior performance across all three datasets, outperforming the other path planning models comprehensively.

**Table 3:** Performance Comparison of the DynaFusion-Plan Model with Other Baseline Models on the TanAir Dataset, NuScenes Dataset and AirSim Dataset.

DataSet	Model	*L*	κ	$R_{\text{OAS}}$	$e_{\text{track}}$	*D*
TanAir	A* [59]	47.3	0.12	92.4	0.18	83
	Dijkstra [60]	45.8	0.14	90.8	0.22	91
	RRT [61]	46.5	0.11	91.2	0.16	95
	Bi-RRT [62]	44.9	0.10	93.1	0.15	88
	PRM [63]	46.0	0.12	91.8	0.17	100
	Hybrid-SLAM [64]	43.7	0.09	94.3	0.14	97
	DynaFusion-Plan	42.5	0.05	98.7	0.12	47
NuScenes	A*	57.6	0.13	89.1	0.22	85
	Dijkstra	56.2	0.14	87.4	0.24	92
	RRT	55.8	0.12	90.2	0.20	94
	Bi-RRT	54.0	0.10	92.0	0.18	87
	PRM	55.0	0.11	91.0	0.19	99
	Hybrid-SLAM	53.5	0.09	93.5	0.16	96
	DynaFusion-Plan	52.3	0.06	97.5	0.11	46
AirSim	A*	41.7	0.14	93.2	0.20	78
	Dijkstra	40.8	0.16	91.6	0.23	90
	RRT	41.2	0.12	92.5	0.18	85
	Bi-RRT	40.5	0.09	94.0	0.16	82
	PRM	41.0	0.11	92.8	0.17	93
	Hybrid-SLAM	39.8	0.08	94.5	0.15	91
	DynaFusion-Plan	38.9	0.04	99.1	0.10	46

In terms of path length, the DynaFusion-Plan model consistently generates significantly shorter paths. On the TartanAir dataset, the path length achieved by DynaFusion-Plan is 42.5 m, representing a 10.1% and 8.6% reduction compared to A* (47.3 m) and RRT (46.5 m), respectively, showcasing the model’s efficiency in path optimization. Similarly, on the NuScenes dataset, the path length of 52.3 m achieved by DynaFusion-Plan is shorter than that of Hybrid-SLAM (53.5 m) and Bi-RRT (54.0 m), further highlighting its exceptional path planning capabilities.

Regarding path smoothness, DynaFusion-Plan also exhibits outstanding performance. Across all datasets, the smoothness metric (κ) of the proposed model is the lowest, indicating that it generates more continuous and smoother paths, facilitating stable vehicle control. For instance, on the AirSim dataset, DynaFusion-Plan achieves a smoothness value of 0.04, outperforming Hybrid-SLAM (0.08) and Bi-RRT (0.09), significantly reducing curvature fluctuations along the path. Additionally, the lower smoothness metric further confirms that the proposed model effectively avoids unnecessary sharp turns or abrupt stops in complex dynamic environments, improving path feasibility.

In terms of obstacle avoidance capability, DynaFusion-Plan significantly outperforms other models in dynamic environments. On the AirSim dataset, the obstacle avoidance success rate of DynaFusion-Plan reaches 99.1%, surpassing A* (93.2%) and PRM (92.8%). Although Hybrid-SLAM achieves a competitive obstacle avoidance success rate in certain scenarios (e.g., 94.3% on the TartanAir dataset), its higher path smoothness metric and tracking errors indicate that it is less robust overall compared to DynaFusion-Plan.

For path tracking accuracy and decision delay, the advantages of DynaFusion-Plan are particularly pronounced. The model achieves the lowest path tracking error and decision delay across all datasets. For example, on the NuScenes dataset, the path tracking error of DynaFusion-Plan is 0.11 m, significantly lower than that of RRT (0.20 m) and Hybrid-SLAM (0.16 m).

In terms of decision delay, DynaFusion-Plan demonstrated significant advantages. On the NuScenes dataset, the decision delay of the model is 44 ms, which is much lower than the traditional algorithms (e. g., PRM and Dijkstra), indicating that the algorithm can quickly respond to environmental changes. In addition, we will further emphasize the real-time path planning capability of the algorithm in practical application, proving its ability to efficiently perform tasks in complex dynamic environment, and provide stable and timely path planning solutions for intelligent logistics unmanned vehicles.

These results validate the potential of the DynaFusion-Plan model as a solution for intelligent logistics path planning. Compared to traditional algorithms (e.g., A* and Dijkstra) and hybrid models (e.g., Hybrid-SLAM and Bi-RRT), DynaFusion-Plan exhibits significant performance advantages, confirming its applicability and superiority in dynamic and complex environments.

**Fig 5:**
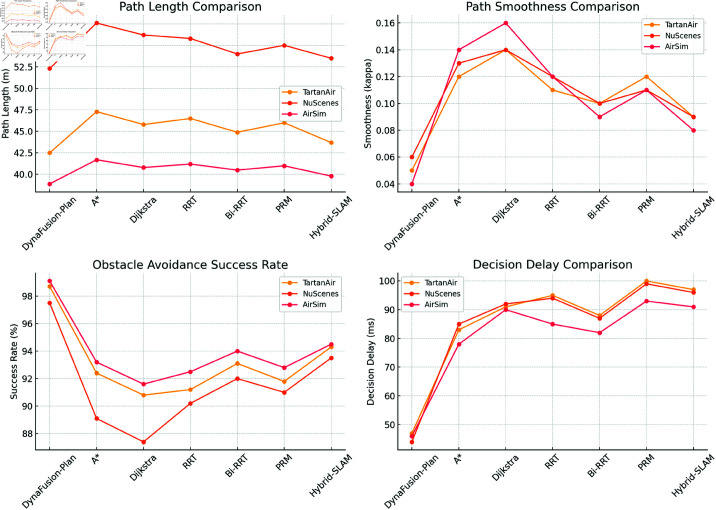
Visualization of Comparative Performance Results Across Datasets for Different Path Planning Models.

Fig 5 provides an intuitive visualization of the performance comparison between the DynaFusion-Plan model and other path planning models across three datasets. The four subplots correspond to the evaluation metrics of path length, path smoothness, obstacle avoidance success rate, and decision delay, respectively. It is evident that the DynaFusion-Plan model excels in all metrics, particularly in path smoothness and obstacle avoidance success rate, showcasing its outstanding capabilities in path optimization and adaptation to dynamic environments. In contrast, traditional models (A* and Dijkstra) exhibit relatively strong path length optimization in simpler scenarios but struggle to balance path smoothness and real-time responsiveness in complex dynamic environments. The visualization results in Fig 5 further validate the comprehensive advantages of the DynaFusion-Plan model.

### Ablation experiment

In the ablation experiments, the study evaluates the performance of the DynaFusion-Plan model across different datasets by sequentially removing the Sensor Data Fusion Module (LIO-SAM), Path Planning Module (APF + DDPG), and Decision and Control Module (MPC + LSTM). The experimental results are summarized in Table 4.

**Table 4:** Ablation Study Results for DynaFusion-Plan Across TartanAir, NuScenes, and AirSim Datasets.

DataSet	Model	*L*	κ	$R_{\text{OAS}}$	$e_{\text{track}}$	*D*
TanAir	Removing Sensor Data Fusion Module (LIO-SAM)	48.7	0.15	85.4	0.25	70
	Removing Path Planning Module (APF + DDPG)	50.2	0.13	79.2	0.30	65
	Removing Decision and Control Module (MPC + LSTM)	45.8	0.09	90.1	0.20	60
	DynaFusion-Plan	42.5	0.05	98.7	0.12	47
NuScenes	Removing Sensor Data Fusion Module (LIO-SAM)	56.4	0.14	83.2	0.23	68
	Removing Path Planning Module (APF + DDPG)	58.6	0.12	75.6	0.28	64
	Removing Decision and Control Module (MPC + LSTM)	54.0	0.1	88.3	0.18	58
	DynaFusion-Plan	52.3	0.06	97.5	0.11	46
AirSim	Removing Sensor Data Fusion Module (LIO-SAM)	45.2	0.12	87.6	0.22	65
	Removing Path Planning Module (APF + DDPG)	46.5	0.11	81.5	0.26	63
	Removing Decision and Control Module (MPC + LSTM)	42.3	0.08	91.7	0.16	55
	DynaFusion-Plan	38.9	0.04	99.1	0.10	46

The comparative results demonstrate that the complete model consistently delivers the best performance across all datasets, validating the importance and rational design of each module in the DynaFusion-Plan architecture. Removing the Sensor Data Fusion Module (LIO-SAM) significantly degrades path smoothness and obstacle avoidance success rate. For instance, on the TartanAir dataset, the smoothness metric increases from 0.05 to 0.15, and the obstacle avoidance success rate drops from 98.7% to 85.4%, highlighting the critical role of multi-sensor fusion in high-precision environmental modeling and dynamic obstacle perception. The impact of removing the Path Planning Module (APF + DDPG) is even more pronounced. On the NuScenes dataset, for example, the obstacle avoidance success rate drops from 97.5% to 75.6%, and the path tracking error increases to 0.28 m, underscoring the core role of the path planning module in optimizing path smoothness and obstacle avoidance capabilities. Removing the Decision and Control Module (MPC + LSTM) leads to a significant increase in decision delay and path tracking error. On the AirSim dataset, for instance, decision delay increases from 46 ms to 55 ms, and path tracking error rises from 0.10 m to 0.16 m, demonstrating the module’s indispensable contribution to real-time path tracking and dynamic adjustments. Overall, the ablation experiments clearly show that the collaborative design of these modules is key to the DynaFusion-Plan model’s superior performance in dynamic environments. This further underscores the rationality and advantages of the proposed model’s architecture.

**Fig 6:**
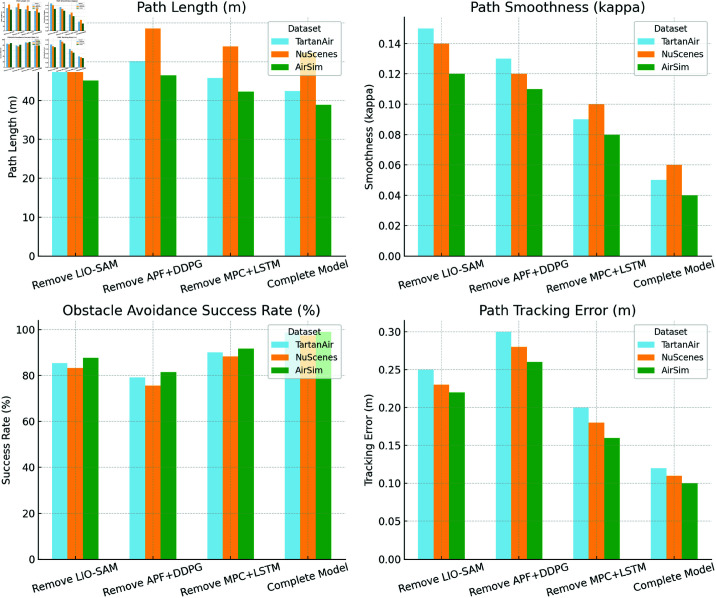
Visualization of Ablation Study Results for Different Model Configurations on TartanAir, NuScenes, and AirSim Datasets.

Fig 6 visualizes the experimental results presented in Table 4, providing an intuitive comparison of different model configurations (complete model and sequentially removed modules) across the TartanAir, NuScenes, and AirSim datasets. The figure highlights that the complete model consistently achieves the best performance across all metrics, particularly excelling in path smoothness and obstacle avoidance success rate. These results further validate the effectiveness of the collaborative design of the model’ s modules.

### Path planning results display

**Fig 7:**
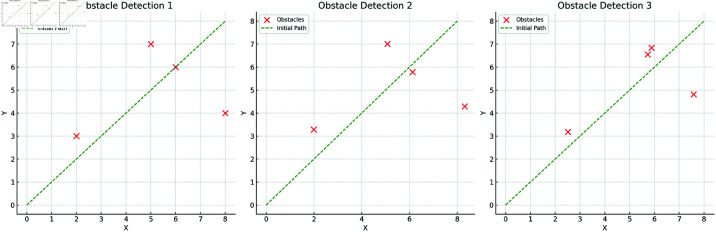
Obstacle Detection and Labeling: Obstacle detection results and initial path labeling in three dynamic environments. It shows how the model accurately detects and labels obstacles in different scenarios, ensuring the effectiveness of subsequent path adjustments.

The experiments validate the model’s efficiency and adaptability in dynamic environments through three key stages: obstacle detection, dynamic path adjustment, and path smoothing optimization. Fig 7 shows the results of obstacle detection, where the obstacle locations may change in dynamic environments. Regardless of how the obstacles are distributed, the model’s sensor fusion module can accurately identify the obstacles and label their positions, providing reliable environmental information for subsequent path adjustment. The differences in obstacle changes and distributions across different dynamic environments also demonstrate the model’s adaptability in diverse scenarios. The results of this phase indicate that the model’s environmental perception capability can effectively adapt to various scene changes.

**Fig 8:**
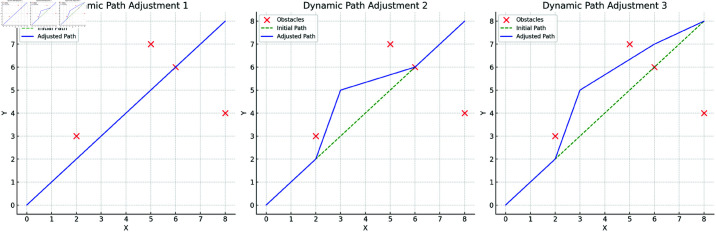
Dynamic Path Planning Adjustment Process: The complete process from initial planning to obstacle avoidance adjustment. It demonstrates how the model dynamically adjusts the path in response to changing obstacles, ensuring safety and reachability.

Fig 8 describes the entire process from the initial planned path to dynamic adjustment. Based on the obstacle detection results, the model can make real-time adjustments to the path according to the distribution of obstacles. In the first adjustment step, the path avoids the main obstacle areas, generating a safe intermediate path. Subsequently, as environmental perception information is further updated, the path is optimized again, ultimately avoiding all obstacles and ensuring path reachability. Dynamic path planning demonstrates how the model effectively adjusts the path based on real-time perception information, showcasing its ability to handle unforeseen situations in complex environments. Through multiple adjustments, the safety and reachability of the path are maximized.

**Fig 9:**
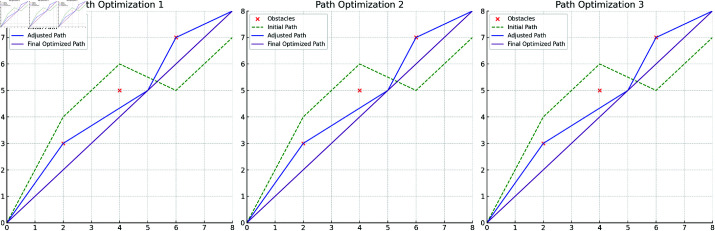
Final Path Smoothing Optimization: Step-by-step results of smoothing optimization applied to the adjusted path. It shows how the path becomes more continuous and efficient through smoothing optimization, enhancing its feasibility and control performance.

Fig 9 shows the effect of the final path smoothing optimization. Based on the adjusted path, the model uses a smoothing optimization algorithm to generate a more continuous final path. The optimized path not only avoids obstacles but also significantly reduces sharp turns and unnecessary complexity, improving the path’s smoothness and driving stability. The smoothed path can be executed more precisely in the autonomous vehicle control system, reducing sharp turns and path fluctuations, thereby optimizing driving stability and energy consumption. Compared to the adjusted path, the smoothed path better meets the needs of autonomous vehicle control, effectively reducing energy consumption and improving driving efficiency. The results in Fig 9 fully validate the model’s comprehensive capabilities in path planning and optimization, laying a solid foundation for practical applications in intelligent logistics scenarios.

Through the results of the three key stages—obstacle detection, dynamic path adjustment, and path smoothing optimization—the model’s path planning capabilities in complex dynamic environments are comprehensively demonstrated, highlighting its advantages in real-time performance, adaptability, and stability.

### Discussion

The experimental results demonstrate that the DynaFusion-Plan model exhibits significant advantages in path planning, dynamic environment adaptation, and path smoothing optimization. First, based on the experimental outcomes and visualizations, the model accurately perceives obstacles in dynamic environments and achieves high-precision environmental modeling through multi-sensor data fusion, providing reliable data support for path planning. The model’s dynamic path adjustment function showcases exceptional real-time performance, enabling it to quickly respond and generate feasible paths even in scenarios with complex obstacle distributions or during path optimization. Moreover, the final smoothed path generated by the model demonstrates high continuity and stability, effectively meeting the driving requirements of autonomous vehicles. However, the results also reveal some limitations. In extreme dynamic environments, such as those with high-density obstacles or rapidly moving obstacles, the efficiency and effectiveness of path adjustments still have room for improvement.

Future optimizations to enhance the model’s robustness and real-time performance in dynamic environments can focus on several aspects. First, incorporating more advanced environmental perception algorithms, such as Transformer-based sensor data fusion methods, could improve obstacle detection accuracy and the precision of environmental modeling details. Second, integrating multi-objective optimization strategies with reinforcement learning methods in the path planning module could better balance multiple metrics, including path length, smoothness, and obstacle avoidance efficiency. Additionally, exploring prediction mechanisms based on dynamic maps could enable the model to anticipate obstacle movement trends, thereby generating more forward-looking path planning solutions. These optimizations would not only further enhance the model’s overall performance but also provide additional practical possibilities for autonomous vehicle path planning in intelligent logistics scenarios.

## Conclusion

The proposed DynaFusion-Plan model demonstrates significant performance advantages in multi-sensor fusion, path planning, and dynamic control. The Sensor Data Fusion Module enables the real-time construction of high-precision dynamic environmental maps, providing reliable perception data for path planning. The Path Planning Module, which combines reinforcement learning with traditional algorithms, optimizes path length and smoothness while achieving efficient obstacle avoidance, particularly in complex dynamic environments. The Decision and Control Module ensures the vehicle’s driving stability and real-time responsiveness through path tracking and dynamic adjustments. The experimental results and visualizations highlight that DynaFusion-Plan achieves superior performance in key metrics, such as path planning efficiency, obstacle avoidance success rate, and path smoothness. Moreover, the model offers robust theoretical and practical support for the application of autonomous vehicles in complex dynamic environments within intelligent logistics scenarios.

Nonetheless, the model has certain limitations, such as high computational complexity and limited applicability in specific scenarios. During multi-sensor data fusion, the model relies heavily on hardware resources, which could restrict its application in resource-constrained environments. Furthermore, the response time for path planning in ultra-high-density obstacle or rapidly changing dynamic environments still requires improvement.

Future research will focus on lightweight model design and optimizing algorithm complexity to reduce computational overhead. Deploying the model in more real-world complex scenarios for validation is another key direction. Additionally, customized optimizations for specific scenarios, such as high-dynamic logistics parks and urban streets, will be an essential focus in future studies. With these enhancements, DynaFusion-Plan has the potential to become a more widely applicable and intelligent path planning solution.
